# Lessons Learned from Donor Cell-Derived Myeloid Neoplasms: Report of Three Cases and Review of the Literature

**DOI:** 10.3390/life12040559

**Published:** 2022-04-08

**Authors:** Komal Galani Deshmukh, Katalin Kelemen

**Affiliations:** 1 Star Superspeciality Hospital, Nagpur 440012, India; drkomalg@gmail.com; 2 Department of Laboratory Medicine and Pathology, Mayo Clinic, Phoenix, AZ 85054, USA

**Keywords:** donor cell-derived leukemia, myeloid neoplasm, hematopoietic stem cell transplantation, therapy-related myeloid neoplasm, cytogenetic findings, bone marrow niche

## Abstract

Donor-cell derived myeloid neoplasm (DDMN), a rare complication after allogeneic hematopoietic cell transplantation (HCT), is of interest for its potential to reveal donor-derived and host-derived factors that contribute to the pathogenesis of leukemia. The accurate diagnosis of donor-derived leukemias has been facilitated by the more frequent use of molecular techniques. In this study, we describe three additional cases of DDMN; the first reported case of donor-derived chronic myelomonocytic leukemia (CMML), one acute myeloid leukemia (AML) with t(8;21)(q22;22); *RUNX1-RUNX1T1* and one donor-derived MDS with deletion 5q. A review of the cytogenetic profiles of previously reported DDMN indicates a significant contribution of therapy-related myeloid neoplasms. Cases with direct evidence of donor- or recipient-dependent factors are rare; a role of direct transfer of leukemic cells, genomic instability of the donor, abnormal gene methylation in donor cells, proleukemic potential of abnormal stromal niche, and the role of immunological surveillance after transplantation has been observed. The role of additional potential pathogenetic factors that are without clinically observed evidence are also reviewed.

## 1. Introduction

Donor-derived myeloid neoplasm (DDMN) is an infrequent complication encountered after allogeneic hematopoietic cell transplantation (HCT) and occurs as a result of the oncogenic transformation of apparently normal donor hematopoietic cells in the transplant recipient. Since the first report of donor-derived leukemia in 1971, an increasing number of cases have been reported in the literature over the past six decades, though a complete catalog of these cases does not exist. Reporting of this intriguing entity has accelerated in recent years due to the more frequent use of molecular techniques. DDMN is the subject of considerable interest for providing unique insights into the mechanisms of donor-derived and host-derived factors of leukemogenesis. In this study, we describe three new cases of DDMN. We analyze the cytogenetic spectrum of previously reported DDMN and discuss individual cases that provide an insight into contributing factors from either the donor’s or the recipient’s perspective. We review the current understanding of DDMN pathogenesis.

## 2. Materials and Methods

### 2.1. Case Selection

Three cases of donor-derived myeloid neoplasm (DDMN) were identified in the Pathology Archives of the Mayo Clinic in Arizona. The diagnosis and subclassification were based on the 2016 World Health Organization Classification of Myeloid Neoplasms [1]. A PUBMED-assisted search of the literature was performed to identify previously reported cases of DDMNs. The morphologic, cytogenetic, interphase fluorescence in situ hybridization (FISH), and molecular genetic findings at original diagnosis and at DDMN are reviewed.

### 2.2. Histologic Evaluation

Peripheral blood (PB) smears were stained with Wright stain. Bone marrow (BM) aspirate smears and biopsy touch imprint preparations were stained with Wright-Giemsa. BM biopsies were fixed in zinc-formalin and stained with hematoxylin eosin (H and E). Evaluation of morphologic dysplasia and blast count was performed on PB and BM aspirate smears. BM cellularity was evaluated on BM core biopsy specimens.

### 2.3. Conventional Cytogenetic and Interphase Fluorescence In Situ Hybridization (FISH) Studies

Karyotype analysis was performed in duplicates on fresh anticoagulated BM aspirate specimens in a complete tissue culture medium. Chromosome preparations, including harvesting and GTW banding, were performed using standard methods. FISH was performed using the Mayo Clinic MDS/AML panel including the following probes and probe strategies: 3q21(*RPN1*), 3q26.2 (*MECOM*) dual color/double fusion; 3q25.32(*MLF1*), 5q35.1(*NPM1*) dual color/double fusion; 5p15.2(*D5S630*), 5q31 (*EGR1*), region gain or loss; 6p23(*DEK*), 9q34(*NUP214*) dual color/double fusion; 7CEN(*D7Z1*), 7q31(*D7S486*) region gain and loss; 8p11.2(*KAT6A*), 16p13.3 (*CREBBP*) dual color/double fusion; 8q22(*RUNX1T1*), 21q22(*RUNX1*), dual color/double fusion; 8CEN(*D8Z2*), 8q24.1(*MYC*), region gain and loss; 9q34(*ABL1*), 22q11.2(*BCR*), dual color/double fusion; 11q23(5′*MLL*[*KMT2A*], 3′*MLL*[*KMT2A*] break-apart; 13q14.3(*D13S319*), 13q34(*LAMP1*), region gain and loss; 15q24.1 (*PML*), 17q21(*RARA*), dual color/double fusion; 16p13(*MYH11*), 16q22(*CBFB*), dual color/double fusion; 17p13(*TP53*), 17CEN(*D17Z1*), region gain and loss; 20q12(*D20S108*), 20qter, region gain and loss. FISH of sex chromosomes was performed using the X centromere (*CEPX*) and Y centromere (*CEPY*) probe set. Cytogenetic abnormalities were classified according to the International System for Human Cytogenetic Nomenclature [2].

### 2.4. BM Chimerism Analysis

Genomic DNA was extracted, and the specimen was evaluated for the percentages of donor and recipient DNA using a PCR-based method that amplifies several highly polymorphic short tandem repeats (STR).

### 2.5. Next Generation Sequencing (NGS) for Hematologic Cancer

DNA was extracted from PB or BM aspirate sample and, following library preparation by hybrid capture, subjected to NGS with post-sequencing analysis of tumor-associated mutations, including the following mutations: *ASXL1*, *BRAF*, *BCOR*, *CALR*, *CBL*, *CEBPA*, *CSF3R*, *DNMT3A*, *ETV6*, *EZH2*, *FLT3*, *GATA1*, *GATA2*, *IDH1*, *IDH2*, *JAK2*, *KIT*, *KRAS*, *MPL*, *MYD88*, *NOTCH1*, *NPM1*, *NRAS*, *PHF6*, *PTPN11*, *RUNX1*, *SETBP1*, *SRSF2*, *TERT*, *TET2*, *TP53*, *U2AF1*, *WT1*, and *ZRSR2*. This NGS panel has a sensitivity of 5–10% variant allele fraction (VAF) with a minimum depth coverage of 250x for single-base substitutions and for insertion/deletion events.

## 3. Results

### 3.1. Case 1

A 71-year-old man was diagnosed with an AML with myelodysplasia-related changes (AML-MRC) characterized by a complex hypodiploid karyotype including deletions of 3p, 5q, and 17p, with one additional metaphase representing a hypo-tetraploid subclone, and a known *TP53* mutation. He was treated with a 7 + 3 induction and mitoxantrone, etoposide, and cytarabine (MEC) chemotherapy, followed by a matched sibling donor PB hematopoietic cell transplantation (HCT) from his brother. He received post-transplant azacitidine and was well until 28 months after transplantation when he developed macrocytic anemia and thrombocytopenia. BM morphologic examination showed 23% small to intermediate-sized blasts with occasional Auer rods and subtle morphologic dysplasia. The cytogenetic study demonstrated t(8;21)(q22;22). The donor, the patient’s 64-year-old brother, was asymptomatic at the time of the onset of DDMN. The donor’s BM biopsy was normal, with a normal male karyotype and no mutations detected by NGS myeloid mutation panel. 

**Figure 1:**
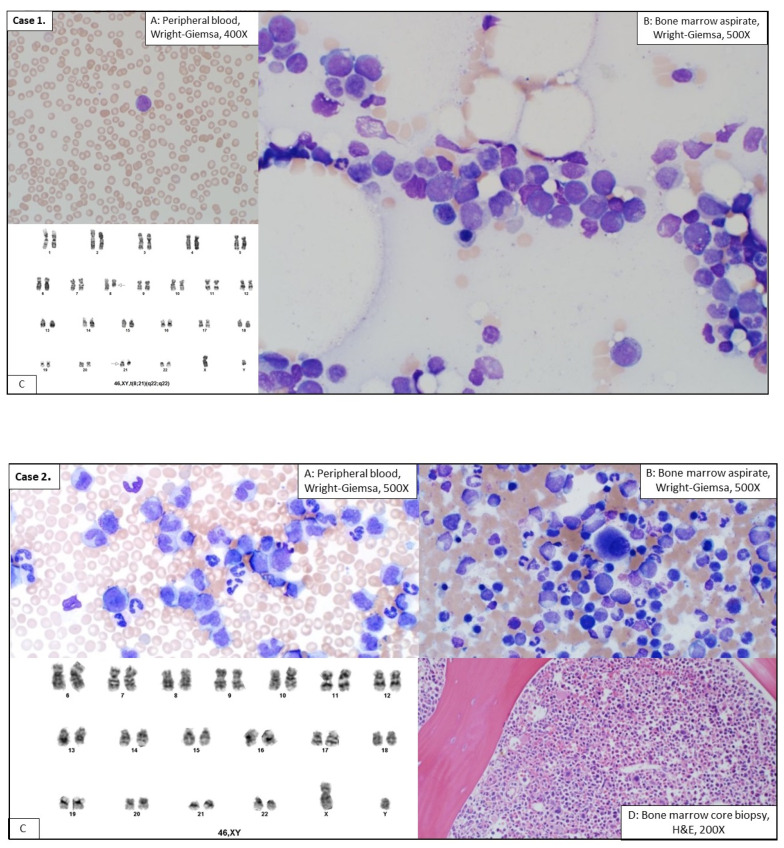

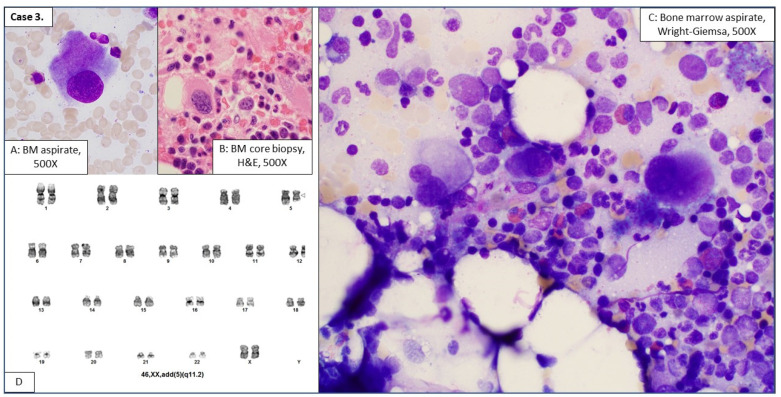
Morphologic and cytogenetic features of three new cases of donor-derived myeloid neoplasm. (**Case 1**), Image (**A**): Peripheral blood smear (Wright-Giemsa, 400X). Pancytopenia and a single circulating blast in the peripheral blood smear of (**Case 1**). Image (**B**): Bone marrow aspirate smear, (Wright-Giemsa, 500X). Numerous blasts seen (23%), meeting criteria of an acute leukemia. Image (**C**): Karyotype analysis shows the presence of t(8:21) (q22;q22). (**Case 2**), Image (**A**): Peripheral blood smear, (Wright-Giemsa, 500X magnification) shows leukocytosis notable for absolute monocytosis (34%, 93,500 K/uL), neutrophilia, basophilia (1%) and granulocytic left shift with 2% circulating blasts. Image (**B**): BM aspirate smear (Wright-Giemsa, 500X) examination showed a cellular marrow with myeloid predominance, 2% blasts, increased monocytes and small monolobated megakaryocytes. Image (**C**): Routine cytogenetic study demonstrated a normal male karyotype. Image (**D**): Bone marrow core biopsy, (H & E, 200X). (**Case 3**), Image (**A**–**C**): Numerous small monolobated megakaryocytes noted in BM aspirate smears (**A**,**C**, Wright-Giemsa, 500X), and in BM core biopsy sections (H and E stain, 500X). In addition, dyserythropoiesis was also noted in the BM aspirate smear. Karyotype analysis shows each of 20 metaphases were 46, XX, and fourteen on them had additional 5q deletion. The 5q region had a segment of unidentified additional material on it, resulting in effective deletion of 5q. Image (**D**): In addition, dyserythropoiesis was also noted in the BM aspirate smear. Karyotype analysis shows each of 20 metaphases were 46, XX, and fourteen on them had additional 5q deletion. The 5q region had a segment of unidentified additional material on it, resulting in effective deletion of 5q.

The patient received 7 + 3 induction and four cycles of high-dose cytarabine chemotherapy. He achieved remission at 7 months after the onset of donor-derived AML, with a normal karyotype and 100% donor chimerism detected in a bone marrow sample. Two years after his donor-derived AML, he developed a relapse of his original AML-MRC, with recurrent complex cytogenetics and *TP53* mutation. He was treated with decitabine and venetoclax, but soon venetoclax had to be withheld due to infectious complications. He died 5 months after the relapsed MDS/AML (58 months after his transplantation). Table 1 summarizes clinicopathologic features and transplant characteristics of cases 1, 2, and 3. Figure 1 presents morphologic and cytogenetic findings of cases 1, 2, and 3.

### 3.2. Case 2

A 59-year-old man with a history of T-lymphoblastic leukemia/lymphoma (T-ALL), status post an allogeneic HSCT from his brother, presented with leukocytosis and splenomegaly 8 years after allo-HSCT. Complete blood counts (CBC) showed hemoglobin of 12.0 g/dL, total leukocyte count of 275,000 cells/μL and platelet count of 316,000/μL. The peripheral blood smear was notable for absolute monocytosis (34%, 93,500 K/μL), neutrophilia, basophilia (1%), and granulocytic left shift with 2% circulating blasts. BM examination showed hypercellular marrow with myeloid predominance, 2% blasts, increased monocytes, and small monolobated megakaryocytes. Flow cytometric analysis was performed on the diagnostic bone marrow aspirate and showed 2% myeloid blasts with an aberrant immunophenotype as follows: CD5, CD13, CD33, CD34, CD117, and HLA-DR positive. Monocytes were increased, comprising 25% of the total analyzed cells and expressed CD13, CD14, CD15, CD33, CD64, HLA-DR, and aberrant CD56. Lymphoid populations showed no immunophenotypic abnormalities.

A routine cytogenetic study demonstrated a normal male karyotype. NGS was positive for mutations of ASXL1, ETNK1, NRAS, and SETBP1. Post-transplant chimerism analysis performed on a bone marrow sample showed 100% donor and 0% recipient DNA. A diagnosis of donor-derived chronic myelomonocytic leukemia (CMML-1) was rendered. The donor was reported healthy at the onset of DDMN. The patient was treated with hydroxyurea. He had a complicated clinical course with polyneuropathy, chronic alcohol dependence, and methicillin-resistant Staphylococcus *aureus* infection, and he died 12 months after the diagnosis of DDMN (Table 1).

### 3.3. Case 3

A 59-year-old male with a history of an allogeneic HSCT 24 years prior to AML presented with macrocytic anemia. The donor was an unrelated matched female. BM showed normocellular bone marrow with many small round megakaryocytes and dyserythropoiesis without an increase in blasts. Flow cytometry performed on the bone marrow aspirate revealed a small myeloid blast population with an unremarkable immunophenotype. Lymphocytes included polyclonal B-cells and T-cells without immunophenotypic abnormalities.

A cytogenetic study performed on a bone marrow sample showed that 20 metaphases were 46, XX, and fourteen of them had additional 5q deletion. The 5q region had a segment of unidentified additional material on it, resulting in the effective deletion of 5q. The diagnosis of a donor cell-derived MDS with deletion 5q was rendered. He was treated with lenalidomide and responded well. The patient is currently alive and well, 32 months after the diagnosis of DDMN. At present, he is off lenalidomide and continues to have mild macrocytic anemia (Table 1).

### 3.4. Summary of DDMN including Current and Previously Reported Cases

A review of the literature identified 82 cases of DDMN, including our current three cases. The demographic, clinicopathological features, and transplant characteristics are summarized in Table S1. These cases represent 56 donor cell-derived AML (including four myeloid sarcomas), 23 donor-derived MDS and three myeloproliferative or myelodysplastic/myeloproliferative neoplasms (MDS/MPN). The most common primary diagnosis to necessitate an allogeneic stem cell transplantation was AML (32.9%), followed by acute lymphoblastic leukemia (ALL) and chronic myeloid leukemia (CML) (19.5% each, respectively), MDS (10.9%) and aplastic anemia (7.3%). Allo-HSCT was less common for lymphomas (3.6%). Rare cases were performed for Langerhans cell histiocytosis or Ewing sarcoma. Occasional DDMNs occurred in recipients of kidney or liver solid organ transplants. More than half of the cases (47 of 82, 57.3%) received a transplant from an opposite-sex donor. Time from allo-HCT to onset of DDMN ranged from 1 to 312 months (Demographic and transplant-related characteristics of 82 cases of DDMN are shown in Table S1). 

### 3.5. Cytogenetic Findings of DDMN

A review of the cytogenetic results revealed that the most common cytogenetic finding in DDMN was a normal karyotype (28.0%), and the most common abnormal karyotype was monosomy 7 (17.2%). Complex karyotypes, MLL rearranged, and t(8;21)(q22;q22) were represented in 6.1% of cases each, respectively, followed by t(15;17)((q24;q21) (4.9%), deletion 5q (4.9%), and the remaining abnormalities were rarely observed, represented by either one or two cases in the cohort (hyperdiploid, inverted 11, trisomy 11, t(1;5), t(3;21), inv(3)(q21;q26), inv(16), t(3;13), t(2;3), t(7;11), add 21, del(20q). In some cases, a cytogenetic result was not reported. Our three new cases represent an AML with t(8;21)(q22;q22), a CMML with a normal karyotype, and an MDS with deletion 5q abnormality. (Cytogenetic findings of 82 cases of DDMN are shown in Table S1). 

### 3.6. Reported Cases of DDMN with Insight into Pathogenesis

Few cases reported findings that provide direct insight into the pathogenesis of DDMN (Table 2). The transfer of a preexisting leukemic clone from the donor to the recipient has been demonstrated in three cases. In one case, the same AML of donor origin with t(1;5) cytogenetic abnormality developed in the donor and in the recipient one month and six months after transplantation, respectively [3]. Preexisting AML cells with Auer rods were present in a smear prepared from the donor bone marrow graft at the time of transplantation. In another case, donor cell-derived AML with trisomy 11 developed in the recipient 14 years after an allo-HCT. In this case, the donor was well and without evidence of AML 14 years after transplant; however, FISH analysis revealed 2% cells with trisomy 11 in a stored blood sample of the donor from the time of donation [4]. In a third case, a female recipient and her male sibling donor developed donor cell-derived AML with inv(3)(q21q26) cytogenetic abnormality at the same time, about 18 months after the allo-HCT [5]. Another potential donor-derived factor is inborn cytogenetic instability of the donor cell, illustrated by a case of AML, MLL rearranged, in a male recipient who received bone marrow grafts from his sister with Bloom syndrome. The recipient received radiation therapy to the testicle after the bone marrow transplant, and that may have contributed to the genomic damage within the donor cells [6]. In another donor cell-derived AML, aberrant p15 gene methylation started to occur on donor cells with normal karyotype 6 months after transplantation, and by 4 years post-transplant, AML developed [7]. The occurrence of donor cell-derived AML in recipients of solid organs, illustrated by two cases after liver transplantation and one case after kidney transplantation [8,9,10], provides evidence that myeloid stem cells reside within solid organs, and outlines the potential contribution of host immunosuppression to the pathogenesis of DDMN. 

## 4. Discussion

Donor cell-derived leukemia (DDL) was first recognized in 1971, but for many years, the paucity of reported cases suggested it to be a rare phenomenon. The majority of evidence in the literature is still anecdotal, and the exact incidence is not known. Based on several reviews, the incidence of DDL has been estimated to be between 0.12% and 5% [15,16,17,18,19]. The diagnosis of donor-derived leukemia is dependent on the ability to accurately identify the donor origin of the leukemic cells. As the cytogenetic and molecular methods evolved from a simple morphological evaluation to karyotype analysis, chimerism, short tandem repeat (STR) analysis by PCR, and more refined molecular biology tools, including NGS, to determine the origin (host versus donor) of normal or abnormal cells in patients posttransplant, the number of reported cases has been increasing. Donor cell leukemias present a unique opportunity to study the events experienced by the hematopoietic stem cells (HSCs) leading to their malignant transformation because HSCT patients are usually followed carefully with repeated bone marrow evaluations. Due to the relatively low number and significant heterogeneity of the documented cases, a single mechanism has not been identified. Donor-derived leukemia is probably a multifactorial process likely following the outline of the so-called multiple “hit” hypothesis in which HSCs suffer a first “hit” in the donor (e.g., an inherited defect of one of the primary caretaker genes), and subsequently are subjected to a second “hit” in an aberrant host environment [20].

In this paper, we report three new cases of DDMN, including a donor cell-derived chronic myelomonocytic leukemia, a subtype that has not been reported before. The other two cases, an AML with t(8;21) and a case of MDS with deletion of 5q, has been reported previously. Only one of the three cases had an opposite-sex transplant. Chimerism analysis has confirmed the donor cell origin of all three cases. Discussion of the myeloid donor cell-derived neoplasms separated from their lymphoid counterparts is justified because myeloid disorders share a cytogenetic classification and often occupy a continuum of disease progression. Since myeloid neoplasms are subclassified along the cytogenetic guidelines, it is informative to review the spectrum of cytogenetic abnormalities in the donor cell-derived cohort. Notably, the single most common cytogenetic affiliation is represented by monosomy 7, an abnormality known to be associated with therapy-related myeloid neoplasm (t-MN) [1]. Monosomy 7, complex karyotypes, and MLL rearranged together represent about one-third of all donor cell-derived MN cases. This suggests a significant contribution of previous therapy received by the recipient, either before, during, or after the allo-HCT, emphasizing similarities to therapy-related MNs. Direct evidence of this mechanism is a donor cell-derived MDS/AML with -7 described by Stevens et al. that developed from the cells of the first transplant after fludarabine and cyclophosphamide conditioning chemotherapy for a second transplant [14]. 

The various hypotheses postulated for the mechanism leading to leukemia usually separate along the lines of donor-derived and recipient-derived factors. Several cases of donor-cell leukemia provide evidence that undetected leukemia cells in the donor at the time of the donation are capable of establishing leukemia in the recipient environment. Accidental transplantation of AML, MDS, CML, cutaneous T-cells lymphoma, and various B-cell lymphomas have been reported [3,4,5,21,22,23,24].

Alternatively, healthy donor HSCs may carry genetic defects predisposing to malignant potential. Since 80% of donor cell leukemias involve related donors, familial cancer predisposition syndromes characterized by germline mutations of tumor suppressor or DNA repair genes, genomic instability, and oncogenic mutations, are of great interest. Hereditary predisposition to MDS/AML has been described with *CEBPA* and *RUNX1* gene mutations [25]. Chromosomal breakage disorders, such as Bloom’s syndrome and Fanconi anemia and Li Fraumeni syndrome (associated with a *TP53* defect), increase the lifetime risk of MDS/AML [26]. In these diseases, a genetic predisposition may be shared between donor and recipient, and the malignant potential is more likely to develop in the posttransplant milieu. An example of this scenario is a case of donor-derived AML, reported by Bielorai et al., where the donor was heterozygous for the Ashkenazi mutation of Bloom’s syndrome [6]. Another case showed evidence of abnormal p15 gene methylation occurring specifically on donor cells, ultimately resulting in a DD-AML [7]. With an unrelated donor, the likelihood of shared predisposition is much less likely. Nevertheless, premalignant clones are well documented in the normal population [27], and latent abnormal clones are particularly common in cord blood donations [28]. Potentially preleukemic clones have been detected by PCR in up to 5% of cord blood samples, including 1% *TEL-AML1*, and 0.2% *AML-ETO* [28], raising the possibility that donor cell leukemia might be more common after cord blood transplantation [29].

Recently there has been a shift in emphasis moving away from the role of the stem cells and toward the microenvironment in understanding the pathogenesis of donor-cell leukemia [30]. Due to the paucity of diagnostic tests that would directly evaluate factors derived from a defective bone marrow stromal niche, this mechanism remains elusive. The BM microenvironment, a complex milieu of fibroblasts, endothelial cells, adipocytes, osteogenic cells, and osteoblasts, provides numerous chemokines and cytokines to contribute to the homeostasis of hematopoietic cells. Though most evidence is indirect, stromal cultures of MDS/AML patients frequently display an abnormal cytokine milieu [31]. The bone marrow stroma plays a crucial role in supporting a hematopoietic cell reconstitution after an allogeneic HSCT, because the donor cells rely on the BM stromal cells of host origin after HSCT [32]. Strong support for the notion that a leukemogenic niche supports DDMN is evident from the case of an MDS patient who developed two clonally distinct donor-cell derived AML after 2 allo-HCTs from a related and an unrelated donor, respectively [11].

In addition, impaired immune surveillance in the recipient microenvironment may promote the evolution of a donor cell neoplasm. T-cells remain quantitatively and qualitatively suppressed for long periods after HSCT [33]. The T-cell reconstitution requires expansion of donor-derived post-thymic T-cells repertoire that has been transferred with the graft. These cells display tolerance to donor cells. A fully competent mature T-cell repertoire with tolerance against both donor and recipient cells requires an adequate thymic function of the recipient, a mechanism that decreases with age [33]. Ultimately, abnormalities of T-cell function can persist for a long time after HSCT. Evidence for suboptimal T-immune surveillance can be derived from the observation that in the early post-transplant period, potentially preleukemic clones may be present, which later disappear spontaneously. Examples of this phenomenon are a case described by Palka et al., with an abnormal clone of 45, XX,-16 at 30 months, that disappeared later [32], and a transient donor-derived MDS with monosomy 7 in a child after cord blood transplantation reported by Sevilla et al. [33]. With the recent advances in molecular platforms that enable screening for preexisting clonal hematopoiesis in donors, evaluation of a relationship between clonal hematopoiesis and DDMN becomes possible, though the associated financial burden still limits a broad application of this approach [11].

Telomere shortening may also have implications in donor-cell leukemia. Telomeres, the noncoding regions of DNA flanking the end of chromosomes, play a critical role in chromosome integrity. Several studies have documented significant telomere shortening following HSCT [34,35,36]. The role of oxygen radicals may contribute to the therapy-related MN-like pathogenesis since it is known that chemotherapy or radiation-induced DNA damage suffered by the stromal cells induces a stress response resulting in the overproduction of reactive oxygen radicals [37]. Experiments from co-culturing HSCs with irradiated BM stromal cells demonstrated that the stromal cells, especially the inflammatory macrophages, are the major source of reactive oxygen radicals [38]. Another possibility is that a dominant oncogen that has been released from residual leukemia cells of the host by either chemotherapy or radiation therapy may directly transfect the donor HSCs. This mechanism could cause residual host disease clones to masquerade as donor cell leukemia. While interesting, this hypothesis does not have direct supporting evidence from a case at this point.

Due to the sporadic nature of reported cases, lack of sufficient follow-up information in many of the cases, and the variations in treatments, it is difficult to assess the prognosis of DDMN in comparison with de novo leukemia. Most cases are reported by case reports of small case series, and at the time of the report, the patient is alive. Overall, the prognosis is predicted to be poor, in line with other secondary leukemias. Based on observations of the case series of European Blood and Marrow Transplantation (EBMT), six of nine patients entered remission after chemotherapy [16]. Long-lasting remission from chemotherapy was observed in a patient with a DDMN of good-prognosis cytogenetic subtype, AML with t(15;17). Five of the nine patients received a second transplant, three of which died, and two were alive at 5 and 11 months after the second transplant, respectively [16]. In general, chemotherapy followed by a second allo-HCT from an alternative donor seems to be a preferable choice of treatment, though a second transplant from the same donor was also applied with success [39], and in some cases, donor lymphocyte infusion resulted in a transient response [40]. A more recent review of donor cell-derived leukemia by Wiseman et al. included outcome data available in 58 patients [19]. Of 58 patients, 34 had died at a median of 5.5 months after the diagnosis of donor-derived leukemia. However, 24 patients were alive at the time of their publication at a median of 14 months follow-up. Median OS (overall survival) for treated patients was estimated at 32.8 months (interval 22.5–43.1). Insufficient data were available for analysis of event-free survival.

Donor-derived leukemias pose a set of ethical questions in relationship to the physician’s responsibility to inform recipients. The afterlife of stem cell donation is not known for unrelated donors, and even sibling donors may not be aware of the clinical outcome in the recipient. Though feedback of adverse events in recipients is encouraged to donor registries, this may be practically not feasible due to the absence of centralization and due to international donations. Further complicating the issue is the observation that most donors do not develop leukemia themselves [16]. The feedback of the adverse outcome may result in a negative impact on the donor’s mindset. Therefore, universal notification would be difficult to justify based on risk. Until further guidelines develop, a discretionary approach may be advised that takes into consideration the wishes of the recipient and the donor if known.

## 5. Conclusions

DDMN is a rare complication of allo-HCT and holds promise as a model to study in vivo leukemogenesis. With the evolution of new molecular techniques for chimerism analysis, the detectability of DDMN became easier. Donor cell-derived neoplasms should be considered for myeloid diseases that develop in the post-transplant period, especially if clinically different from the original disease. The etiology is multifactorial, and the relative contribution of each of the genetic and transplant-related factors is still largely unknown. Further molecular and immunological investigations would help clarify the pathogenesis. There is a need for large multicentric studies to ensure more inclusive reporting and comprehensive analysis of cases.

## Figures and Tables

**Table 1:** Summary of three new cases of donor-derived myeloid neoplasm.

	CASE 1	CASE 2	CASE 3
**Age/Gender**	71 y/Male	59/Male	59/Male
**Original** **diagnosis**	AML with MDS-related changes	T-ALL	AML, NOS
**Cytogenetic** **findings**	Complex hypodiploid with deletions of 3p, 5q and 17p and a hypotetraploid subclone	//46,XY	//46,XY
**Molecular findings**	*TP53* mutation	Not tested	Not tested
**Donor type and gender**	Matched sibling Male	Matched sibling Male	Matched unrelated Female
**HSCT**	PBSC	PBSC	PBSC
**Time to DDMN** **(Months)**	28	96	288
**DDMN** **type**	AML with t(8;21)	CMML-1	MDS with 5q-
**DDMN karyotype**	46,XY,t(8;21)(q22;q22) [12]	//46,XY [46]	//46,XX,add(5)(q11.2) [14]/46,XX [6]
**Chimerism** **(BM)**	95% donor, 5% recipient	100% donor	100% donor
**DDMN molecular findings**	None	*ASXL1*, *ETNK1*, *NRAS* and *SETBP1* mutated	None

**Table 2:** Donor-derived and host-derived factors in reported cases of DDMN.

DONOR-DERIVED FACTORS		
Transfer of preexisting leukemia	Evidence	References
**DD-AML with t(1;5) developed in the donor and recipient**	AML cells with Auer rods present in the BM graft smear at time of donation	[3]
**DD-AML with trisomy 11 in the recipient 14 years after allo-HCT. Donor without evidence of AML.**	Preleukemic cells with trisomy 11 in a stored blood sample of donor at time of donation (2% by FISH)	[4]
**DD-AML with inv(3)(q21q26) in recipient and donor at the same time**	Donor harbors preexisting cells with inv(3)(q21q26)	[5]
**Inborn genetic abnormality of donor**		
**DD-AML, MLL rearranged**	Donor heterozygous for the Ashkenazi mutation of Bloom’s syndrome. Recipient received radiation after allo-HCT.	[6]
**DD-AML, normal karyotype and aberrant p15 gene methylation.**	Aberrant p15 methylation on donor cells at 6 months after allo-HCT. DD-AML developed by 4 years. P53 gene mutation was absent. Donor developed bronchogenic carcinoma.	[7]
**HOST-DERIVED FACTORS**		
**Leukemogenic BM niche**	Two distinct DD-AML developed in same host after two allo-HCT. Original diagnosis of MDS with del(7)del(20q). 18 months after first allo-HCT DD-AML with t(8;21). Sixty months after second allo-HCT DD-AML with normal karyotype.	[11]
**Role of recipient immune surveillance: Transient donor derived clones in recipient**	45, XX,-16 donor clone present at 30 months after allo-HCT. Monosomy 7 donor clone in a child 3 months after allo-HCT.	[12,13]
**Therapy-related myeloid neoplasm**	DD MDS/AML with -7 from the cells of the first transplant after fludarabine and cyclophosphamide conditioning chemotherapy for a second transplant.	[14]

## Data Availability

Not applicable.

## Supplementary Materials

The following supporting information can be downloaded at: https://www.mdpi.com/article/10.3390/life12040559/s1, Table S1: Demographic and transplant-related characteristics of 82 cases of DDMN [41,42,43,44,45,46,47,48,49,50,51,52,53,54,55,56,57,58,59,60,61,62,63,64,65,66,67,68,69,70,71].
