# Supplementary material for: Lessons Learned from Donor Cell-Derived Myeloid Neoplasms: Report of Three Cases and Review of the Literature
Source: Life (Basel). 2022 Apr 8;12(4):559. doi: 10.3390/life12040559 (PMC9028156; doi:10.3390/life12040559)
Supplement: Supplementary file 1 [file life-12-00559-s001.zip › life-1637666-supplementary.pdf]

life

MDPI

Case Report

Lessons Learned from Donor Cell-Derived Myeloid Neoplasms. Report of Three Cases and Review of the Literature

Komal Galani Desmukh [1] and Katalin Kelemen [2,*]

Supplementary Materials:

Table S1. Demographic and transplant-related characteristics of 82 cases of DDMN.

	Author Year Reference	Original diagnosis	Original Cytogenetic finding	Age/gender of host at HCT	Time to DDMN (Mo)	D sex	DDMN	DDMN Cytogenetic finding	Donor Factors	Recipient Factors
					AML cases					
**1**	Goh, 1977 [41]	AML	U	6y/F	21	M	AML	XY, hyperdiploid with additional unknown material	46,XY	N/A
**2**	Elfenbein, 1978 [42]	AML	U	22y/M	34	F	AML t(8;21)	45,XX,t(8;21) (q22;q22)	46,XY	N/A
**3**	Witherspoon, 1985 [43]	ALL	U	25y/F	72	F	AML	XX, complex karyotype	46,XX	N/A
**4**	Niederwieser 1990 [3]	CML	46,XX,t(9;22)	35y/F	1	M	AML	46,XY,t(1;5)	AML with t(1;5)	N/A
**5**	Browne, 1991 [44]	AA	46,XY	19y/M	9	F	AML	XX,t(9;11)(p22;q23)	46,XX	N/A
**6**	Lowsky, 1996 [45]	CML	46,XY,t(9;22)	46/M	24	F	AML	45,XX,-7	46,XX	N/A
**7**	Bodo, 1999 [8]	PBC	U	55/F	24	M	APL	46,XY, t(15;17) HLA-type of donor	46,XX	Immuno-suppression
**8**	Spinelli, 2000 [46]	ALL, Ph+	46,XY,t(9;22)	49/M	26	F	AML	47,XX, del(9) (p21), +i(17) (q10), del(20) 47,(q12), ider(22) (qter), t(9;22) (q34;q11)	46,XX	N/A
**9**	Cooley, 2000 [47]	CML	46,XY,t(9;22)	Unknown /M	131	F	AML t(8;21)	45,X,t(8;21)[12]/45,XX[3]	46,XX	N/A
**10**	Hambach, 2001 [48]	CML	46,XY,t(9;22)	38/M	14	M	AML	46,XY	46,XY	N/A
**11**	Lawler, 2002 [49]	AA	46,XX	12/F	5.7	F	AML	46,XX STR of donor origin	46,XX,	N/A

**12**	Au, 2002 [7]	CML	46,XX, t(9;22)	47/F	48	M	AML	46,XY	46,XY	Aberrant p15 methylation
**13**	Gopcsa, 2002 [50]	CML	46,XY, t(9;22)	54/M	60	F	AML	46,XX	46,XX	N/A
**14**	Bielorai, 2003 [6]	ALL	Unknown		48	F	AML	46,XX, inv(11)(p15q23)	46,XX, Bloom syndrome	Radiation to testicles 2 years after HCT
**15**	Haltrich, 2003 [51]	AA	Unknown	2.5/F	156	M	AML	46,XY, t(3;21)(q26;q21)	46,XY	N/A
**16**	Subklewe, 2004 [9]	Cirrhosis	Unknown	43/M	36	NR	AML	Complex,-18, r(5), +13, others, STR 100% donor, HLA donor type	Unknown	Immuno-suppression
**17**	Hertenstein, 2005 [12]	ALL	46,XX	24/F	15	M	AML t(8;21)	46,XY, t(8;21)	46,XX	N/A
**18**	Hertenstein, 2005 [12]	AA	46,XY	21/M	9	F	AML	46,XX,t(9;11)	46,XX	N/A
**19**	Hertenstein, 2005 [12]	AA	46,XX	12/F	19	F	AML	46,XX, STR of donor	46,XX	N/A
**20**	Hertenstein, 2005 [12]	AML	46,XX	35/F	5	M	AML	46,XY, add(3)del(12_	46,XY	N/A
**21**	Hertenstein, 2005 [12]	AML	46,XY	34/M	39	F	AML	46,XX	46,XX	N/A
**22**	Hertenstein, 2005 [12]	AML	46,XX	47/F	78	F	AML	46,XX, STR of donor	46,XX	N/A
**23**	Hertenstein, 2005 [12]	CML	46,XY,t(9;22)	40/M	14	M	AML	46,XY	46,XY	N/A
**24**	Hertenstein, 2005 [12]	CML blast crisis	46,XY,t(9;22)	35/M	39	M	AML	46,XY,t(15;17)	46,XY	N/A
**25**	Hertenstein, 2005 [12]	CML	46,XX,t(9;22)	56/F	67	M	AML	42,XY,-6,-7,-11, der(17),-18	46,XY	N/A
**26**	Ando, 2006 [52]	AML	47,XX, add(10)(p11)-11,?13p,+mar1 [20]	32/F	11	F	AML	46,XX 100% donor STR	46,XX	N/A
**27**	Pieroni, 2006 [53]	CML	46,XX,t(9;22)	45/M	52.8	M	AML	46,XY,del(6)(q23q25), inv(16)(p13q22)	46,XY	N/A
**28**	Reichard, 2006 [54]	T-ALL	46,XY,t(10;14)(q24;q11.2), with	33/M	16	F	AML	46,XX,11q23 rearranged	46,XX	N/A

			abnormalities of 6 and 8							
**29**	Sala-Torra, 2006 [21]	RCC	N/A	40/M	15	M	AML	46,XY	46,XY	N/A
**30**	Jacobs, 2007 [39]	B-ALL	46,XY	1/M	48	M	AML	46,XY,t(9;11)9p22;q23)	46,XY	N/A
**31**	Nagamura-Inoue, 2007 [55]	AML-ETO	46,XX,t(8;21)(q22;q22.1)	32/F	15	M	AML,	46, XY, 100% donor STR	46, XY	N/A
**32**	Nagamura-Inoue, 2007 [55]	AML, NOS	46,XX	32/F	11	F	AML	46,XX, 100% donor STR	46,XX	N/A
**33**	Nagamura-Inoue, 2007 [55]	ATL, HTLV+	46,XX	56/F	7	M	AML	46,XY, 100% donor STR, HTLV-	46,XY	N/A
**34**	Nagamura-Inoue, 2007 [55]	HL	46,XY	30/M	16	F	AML	46,XX, MLL rearranged	46,XX	N/A
**35**	Glasser, 2009 [5]	AML	46,XX,t(3;5)(q25;q34)	42/F	21	M		46,XY,inv(3)(q21q26)	46,XY 1y after the DDMN the donor developed AML with same CG as DDMN	
**36**	Crow, 2010 [56]	AML	46,XX, t(11;19)(q23;p13)	3/F	14	M	AML	45,XY,-7	46,XY	N/A
**37**	Wang, 2011 [14]	AML	46,XX, t(11;19)(q23;p13.3)	3/F	14.5	M	AML	45,XY,-7[18]/46,XY[1]	46,XY	N/A
**38**	Wang, 2011 [14]	CML	46,XY,t(9;22)(q34;q11.2)	58/M	193	F	AML	46,XX	46,XX	N/A
**39**	Otero, 2021 [57]	AA	46,XY	23/M	33	F	AML	45,XX,-7	46,XX	N/A
**40**	Dickson, 2014 [4]	t-AML, inv(16)	46,XY,inv(16)9p13q22)	43/M	168	M	AML	47,XY,+11	Trisomy 11 in PB at the time of donation	N/A No AML develops in donor
**41**	Dietz, 2014 [58]	LHC	Unknown	1/Unknown	42	NR	AML	Unknown	Unknown	N/A
**42**	Dietz, 2014 [58]	FA	Unknown	11/Unknown	16	NR	AML	Unknown	Unknown	N/A
**43**	Dietz, 2014 [58]	MDS	Unknown	47/Unknown	60	NR	AML	Unknown	Unknown	N/A
**44**	Girsberger, 2013 [10]	Polycystic kidney	Unknown	69/M	24	F	AML	46,XX, *NPM1* mutated	Kidney donor	N/A

**45**	Shiozaki, 2014 [59]	AML-MRC	Complex,47,XY, +8[11],47,XY, del(2)(q21q31)+8[5], others	53/M	24	F	AML-MRC	46,XX, r(7)(p10p22)[26]/46, XX[5}99.8% XX by FISH 100% donor STR	46,XX	N/A
**46**	Aldoss, 2017 [60]	ALL	46,XY,t(5;14)(q31;q32)	32/M	15	46/F	AML	46,XX,t(3;13)(p10;q10)	46,XX	N/A
**47**	Aldoss, 2020 [30]	MDS	46,XX, del(7)(q11.2q32)	60/F	18	54/M	AML	46,XY,t(8;21)(q22;q22)[2],45,sl,Y[7],46,sl, del(9)(q13q22)[2]46, XY[10]	46,XY	N/A
	Second transplant				60	32/M	AML	46,XY		
**48**	Bouvier, 2018 [61]	AML	46,XX	50/F	36	F	APL	46,XX,t(15;17) 100% DONOR CHIM	46,XX	N/A
**49**	Bouvier, 2018 [61]	AML	46,XY	25/M	279 mo	F	APL	47,XX,+8,t(15;17)(q24;q21)	46,XX	N/A
**50**	Suarez-Gonzalez 2018 [62]	B-ALL	46,XX, t(1;19)	43/F	16	F	AML, *NPM1* mutated	46,XX	46,XX	N/A
**51**	Galani, 2021 Current paper	AML-MRC	Complex hypodiploid karyotype, *TP53* mutation	71/M	28	M	AML	46,XX,t(8;21)(q22;q22)	46,XY	N/A
					**AML, Myeloid sarcoma cases**					
**52**	Jeong, 2010 [63]	AML	46,XY	35/M	57	M	MS, duodenum, jejunum, skeletal muscle	Duodenum: 46,XY, donor origin Bone marrow: 100% donor, no leukemia	46,XY	N/A
**53**	Walshauser, 2014 [64]	MDS	45,XY,der(5)t(5;7)9q31;q22),add(11)(p15)	56/M	23	F	MS Stomach	Stomach tissue XX by FISH; 100% donor chimerism in blood	46,XX	N/A
**54**	Otsuji, 2021 [65]	AML	46,XY,t(6;11)(q27;q23)	49/M	60	F	MS, right leg bone	95% XX by FISH 20q-, 17q-, gains of 5, 6, 8, 15q, BM 46,XX, 20q-	46,XX	N/A
**55**	Otsuji, 2021 [65]	AML-MRC	47,XY,+8/second clone with +6,+8,+12,+21	40/M	84	F	MS testis	XX by FISH BM negative	46,XX	N/A
					**Myelodysplastic syndrome cases**					

**1**	Brunstein, 2002 [66]	CLL and Th-MDS	45 XY,-7 46,XY,del(20q) (q11.2q13.3)	41/M	42	M	AML	46,XY, add(21)(q22)	46,XY,	N/A
**2**	Komeno, 2003 [40]	MDS	47,XY,+8,del(20)(q11)	46/M	24	F	AML	47,XX,+11	46,XX	N/A
**3**	Sala-Torra, 2006 [21]	CML	46,XY,t(9;22)	48/M	48	M	MDS	45,XY,-7	46,XY	N/A
**4**	Sala-Torra, 2006 [21]	CML	46,XX,t(9;22)	33/F	48	F	MDS	45,XX,-7	46,XX	N/A
**5**	Sala-Torra, 2006 [21]	CML	46,XY,t(9;22)	45/M	36	F	MDS	46,XX,del(20q)	46,XX	N/A
**6**	Sala-Torra, 2006 [21]	AA/ PNH	46,XY	11/M	312	F	MDS	46,XX,del(5q)	46,XX	N/A
**7**	Sala-Torra, 2006 [21]	ALL	46,XY	4/M	48	F	MDS	46,XX,inv(11)	46,XX	N/A
**8**	Hashino, 2006 [67]	AA	46,XX	23/F	31	F	MDS	45,XX,t(2;3),-7	46,XX	N/A
**9**	Glasser, 2009 [5]	MDS-EB2	46,XX,t(3;5) (q25;q34)	42/F	18	M	MDS-EB2	45,XY,inv(3)(q21q26),-7	45,XY,inv(3) (q21q26),-7 Clone transfer	N/A
**10**	Wang, 2011 [14]	ALCL, t-MDS	46,XY	53/M	82	M	MDS-MLD	45,XY,-7[6]/45,XY, idem,r(6)(9p23q25)[11]	46,XY	N/A
**11**	Wang, 2011 [14]	MCL	46,XY,t(11;14)(q13;q32)	58/M	22	F	MDS-U	46,XX, del(20)(q11.2q13.1)	46,XX	N/A
**12**	Wang, 2011 [14]	CLL, t-MDS	45,XY,-7 9.5% by FISH	55/M	6	M	MDS-U	Monosomy 7 by FISH, 68%	46,XY	N/A
**13**	Wang, 2011 [14]	CML	46,XX,t(9;22)	66/F	166	M	MDS-U	46,XY,-7[19]	46,XY	N/A
**14**	Wang, 2011 [14]	B-ALL	46,XY,t(17;19)	44/M	26	F	MDS-U	46,XX,del(7)(q22q32)[2]46,XX[27]	46,XY	N/A
**15**	Wang, 2011 [14]	B-ALL	45~49,XX,add(12p)del(13q),i(17q), others	22/F	5	M	MDS-U	45,XY,-7[8]46,XY[13]	46,XY	N/A
**16**	Wang, 2011 [14]	AML	46,XY,inv(7)(q22q34)	70/M	1	F	MDS-SLD	46,XX,del(5)(q13q31)[4]46,XX[15]	46,XX	N/A
**17**	Shah, 2012 [68]	Ewing sarcoma	46,XX	25/F	84	F	MDS-EB2	46,XX, 100% donor STR	46,XX	N/A
**18**	Dietz, 2014 [58]	AML	NR	11/NR	19	NR	MDS	NR	Unknown	N/A
**19**	Dietz, 2014 [58]	AML	NR	38/NR	3	NR	MDS	NR	Unknown	N/A

**20**	Dietz, 2014 [58]	MDS/AML	NR	57/NR	1	NR	MDS	NR	Unknown	N/A
**21**	Schwartz, 2018 [69]	B-ALL, Ph-like	46,XY, t(2;9)(q21;q34)	15/M	66	43/F	MDS	45,XX,-7, 100% CHIM *ASXL1* mutated	46,XX	N/A
**22**	Zhang, 2021 [70]	MDS-RAEB2	46,XY	46/M	156	M	MDS-EB2	46,XY, 100 donor STR *DNMT3A*+, *TET2*+	46,XY	N/A
**23**	Galani, 2021 Current paper	AML, NOS	46,XY	59/M	288	F	MDS with 5q-	//46,XX,add(5)(q11.2)[14]/46,XX[6]	46,XX	N/A
	**Myeloproliferative neoplasm or myelodysplastic/myeloproliferative neoplasm cases**									
**1**	Mitsui, 2007 [71]	B-ALL, Ph+	46,XY,t(9;22)/46,XY,inv(9p)	41/M	9	F	MPN	46,XX,t97;11)(p15;p15)	46,XX	N/A
**2**	Hertenstein, 2005 [12]	CML blast crisis	46,XX,t(9;22)	25/F	4	M	CML	46,XY,t(9;22)	46,XY	N/A
**3**	Galani, 2021 Current paper	T-ALL	46,XY	51/M		M	CMML	46,XY 100% donor chimerism *ASXL1*, *ETNK1*, *NRAS* and *SETBP1* mutations	46,XY	N/A

Abbreviations: HCT: hematopoietic cell transplantation, Mo: months, D: donor, AML: acute myeloid leukemia, AML-MRC: acute myeloid leukemia with myelodysplasia-related changes, U: unknown, Y: year, F: female, M: male, N/A: not applicable, NR: not reported, MDS: myelodysplastic syndrome, MDS-EB1: myelodysplastic syndrome with excess blasts 1, ALL: acute lymphoblastic leukemia/lymphoma, AA: aplastic anemia, FA: Fanconi anemia, PNH: paroxysmal nocturnal hemoglobinuria, HL: Hodgkin lymphoma, ATL: Adult T-cell leukemia/lymphoma, CML: chronic myeloid leukemia, CMML: chronic myelomonocytic leukemia, MS: myeloid sarcoma, CLL: chronic lymphocytic leukemia, DDMN: donor-derived myeloid leukemia, CG: cytogenetics, STR: short tandem repeats, RCC: renal cell carcinoma, LHC: Langerhans cell histiocytosis, PBC: primary biliary cirrhosis.
